# Novel compound heterozygote mutations in the *ATP7B* gene in an Iranian family with Wilson disease: a case report

**DOI:** 10.1186/s13256-018-1608-0

**Published:** 2018-03-15

**Authors:** Omid Daneshjoo, Masoud Garshasbi

**Affiliations:** 1 Department of Molecular and Cell Biology, Nano and Biotechnology Research Group, Faculty of Basic Sciences, University of Mazandaran, Babolsar, Iran; 2 Department of Medical Genetics, Faculty of Medical Sciences, Tarbiat Modares University, Tehran, Iran; 3 Medical Genetics Department, DeNA laboratory, Tehran, Iran

**Keywords:** Wilson disease, Compound heterozygote, Sequencing, *ATP7B* gene

## Abstract

**Background:**

Wilson disease is an autosomal recessive disorder of copper transport and is characterized by excessive accumulation of cellular copper in the liver and other tissues because of impaired biliary copper excretion and disturbed incorporation of copper into ceruloplasmin. Hepatic failure and neuronal degeneration are the major symptoms of Wilson disease. Mutations in the *ATP7B* gene are the major cause of Wilson disease.

**Case presentation:**

In this study we have screened one pedigree with several affected members, including a 24-year-old Iranian woman and a 20-year-old Iranian man, who showed psychiatric and neurological symptoms of varying severity, by amplifying the coding regions including exon–intron boundaries with polymerase chain reaction and sequencing. We identified c.1924G>C and c.3809A>G mutations in affected members as compound heterozygote state. These mutations segregated with the disease in the family and they were absent in a cohort of 100 Iranian ethnicity-matched healthy controls.

**Conclusions:**

No homozygote state has been reported for these two variants in public databases. *In silico* predicting tools consider these two variants to be damaging. So this study introduces the novel combination of c.1924G>C and c.3809A>G variants as a cause for Wilson disease.

## Background

Wilson disease (WD) is a copper metabolism disorder causing injuries in several tissues like the cornea of the eye, the brain, and liver [1], which was initially described by a British neurologist S. A. K. Wilson as progressive lenticular degeneration [2]. A Kayser–Fleischer ring [3] in the cornea of the eye is a hallmark of WD. The disease incidence is 1 in 35,000–100,000 in live births [4].

WD has an autosomal recessive (AR) mode of inheritance and occurs due to a deficiency in one of the ATPase cu^2+^ cellular pumps called ATP7B which is coded by the *ATP7B* gene. This gene is located on chromosome 13q14.3 with 78,826 bp length and consists of 21 exons [5]. *The ATP7B* gene codes the ATP7B protein, which is an acronym for: ATPase activity, 7 distinct domain, and B class for second P-type ATPase copper binding pump. This molecule interacts with several other molecules such as ATOX1, ATP7A, CTR1, and DMT1 depending on the cell type [3].

WD is a disease with extensive clinical heterogeneity and largely nonspecific symptoms. The age of onset varies from age 8 to 50. Patients with WD usually are diagnosed in their early teens as having some form of hepatic dysfunction that leads to decreased biliary excretion and elevated excretion of urinary copper [6]. A small proportion of patients with WD (approximately 20%) develop bone and joint disorders. The patients can be divided into three major groups based on their disease outcomes: those displaying hepatic symptoms, those displaying neurological symptoms, and, finally, those displaying both hepatic and neurological symptoms [7]. In patients with mainly neurologic outcomes serum pentraxin-3 is elevated [8]. The last stage of the disease involves accumulation of copper in organs such as the brain, kidney, and cornea [9].

Therapeutic activities against this disease mainly consist of using chelating agents like penicillamine and trientine to reduce the amounts of accumulated copper in the body [10].

More than 400 [11] different mutations consisting of deletions, insertions, duplications, and substitutions which can cause missense, nonsense, frameshift, splice site mutation, and *Alu* exonization in *ATP7B* gene transcription have been reported which lead to relatively different clinical features [3, 12–14]. The rate of these mutations varies in different subpopulations [15–17]. The majority of WD mutations (~ 60%) are of the missense type [18] and the commonest point mutation in WD is His1069Glu [6], which is presented in approximately 38% of the genome of all patients with WD from North American, Russian, and Swedish samples [19].

In this study we screened *ATP7B* gene in six members of an Iranian family with multiple affected members suspected to have WD to find the mutation which causes the illness. This study resulted in the identification of a novel combination of two mutations in *ATP7B* gene in affected members of this family (ClinVar Submission ID: SUB2992698).

## Case presentation

**Fig. 1:**
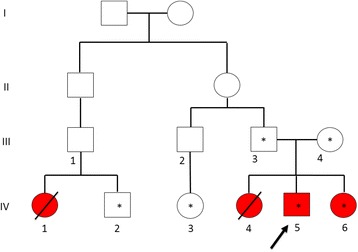
Pedigree of an Iranian family with Wilson disease. *Red shading* denotes affected individuals. Circles and squares with * indicate individuals who were analyzed by Sanger sequencing. The arrow in the pedigree shows the proband (the first sample in the family who was referred to us and was studied)

The pedigree studied here consisted of four affected members with similar clinical features of whom only two were alive at the time of this report (Fig. 1).

All participants, or their legal guardians, provided written and informed consent. The institutional review boards of Tarbiat Modares and Mazandaran University reviewed the project.

A 24-years-old Iranian woman (member IV:5) and a 20-years-old Iranian man (member IV:6) showed psychiatric and neurological symptoms of varying severity. These symptoms included ataxia, tremor, dystonia, dysarthria in speech, and weak mental state. Their ceruloplasmin range was elevated and the Kayser–Fleischer ring was present in both of them. DNA was extracted from their peripheral blood (IV:5 and IV:6), from their parents (III:3 and III:4), and from two healthy family members (IV:2 and IV:3) using standard protocols.

Table 1 depicts the sequence of primers used for amplifying the whole coding region and exon–intron boundaries of the *ATP7B* gene. Primers were designed using Primer 3 software (version 0.4.0). Primers specificity was checked by *in silico* polymerase chain reaction (PCR) and blat tools of University of California, Santa Cruz (UCSC) genome browser, and lack of single nucleotide polymorphisms (SNPs) in the genomic region corresponding to the 3′ ends of the primers was checked by looking through the Single Nucleotide Polymorphism database (dbSNP).

**Table 1:** Primer sequences used for polymerase chain reaction amplification of all exons and exon–intron boundaries of *ATP7B* gene

Exon 1	F5′-CCGGTCCCAAATGAAGG-3′ R5′-TTTTCTCCCACGCCAAG-3′
First part of exon 2	F5′-CAGAGAAGCTGGGATGTTGTAG-3′ R5′-GATAAAGGTCTCTTTGGGTTAGTG-3′
Second part of exon 2	F5′-TTTGAAGCTGCCATCAAGAG-3′ R5′-GACACAAAGAGAAAAGGAGACAAG-3′
Exon 3	F5′-GACAATGAACCCTCACCAAG-3′ R5′-ACTGAGAAGTCTATCCACAAAAGG-3′
Exon 4	F5′-GGGAAGATGTGTTTCTTTGTTC-3′ R5′-CACCGTCTTTAATTCCTGTTTC-3′
Exon 5	F5′-CTGTTGCCATCTGCTTCAC-3′ R5′-CTCATCTTTCTCTTACCCATTCAC-3′
Exon 6	F5′-TCTACTGAGGCACTTTTAGATTCAC-3′ R5′-CTGTTTCAGAGGGTTCACATTAC-3′
Exon 7	F5′-GCAGGTCTTAAACTGTGTCCTC-3′ R5′-GGTGATCCAGTTGTTGCTTC-3′
Exon 8	F5′-GACTGTGCACAAAGCTAGAGG-3′ R5′-CTAAACATGGTGTTCAGAGGAAG-3′
Exon 9	F5′-CAGTGGGAAGACTGATGTTTG-3′ R5′-GTTCTCTGTGAAGTTTCCCTTG-3′
Exon 10	F5′-CATTTCTACCACAGAACTTGTCTTC-3′ R5′-TTGACATCTGAGCCTCTTCC-3′
Exon 11	F5′-GATGGCTTGTTTCATGTTCC-3′ R5′-CTGATTTCCCAGAACTCTTCAC-3′
Exon 12	F5′-GTAATTGCGGGGTCTATAAATG-3′ R5′-TAATAGAAACCTGCAGAAGGAGAG-3′
Exon 13	F5′-CCTCTGACTCTGTCCTGTTTTC-3′ R5′-TTGGTCAAGTTACCTAATCTCCTC-3′
Exon 14	F5′-ATCTGTATTGTGGTCAGTGAGTTG-3′ R5′-TAGGAGAGAAGGACATGGTGAG-3′
Exon 15	F5′-GCTTACAGTTTCCTCTTCCTCTC-3′ R5′-AATTTAGACGCACCCAAGAAC-3′
Exon 16	F5′-CAAATACCTGAGTGCTTCTAATCC-3′ R5′-GGAAGGCTTTTGTTTGTCTTC-3′
Exon 17	F5′-TTCTGCAGGAAAAGACGAAG-3′ R5′-ATCCAGCAAGGGAGAAAGAG-3′
Exon 18	F5′-ATGTGAAGCAGGAGAGTAGGG-3′ R5′-AGCAAATCATTCTGATGGAGAG-3′
Exon 19	F5′-ACTGTGTGCTCCTCTCCATC-3′ R5′-GTCAAAGAGCCATTTCTTTCC-3′
Exon 20	F5′-GAGCTCGCCCTGAAATG-3′ R5′-TGTCCCAGGTGAATGAATG-3′
Exon 21	F5′-CTCAGATGCTGTTGCGTTC-3′ R5′-TCACAGCAGTCATCCTAAATACTC-3′

PCR analysis was carried out in a total volume of 25 μl containing 0.5 μl of each forward and reverse primers (10 Pmol), 10 μl of PCR Master mix Mgcl2 1.5 mM and 1 μl DNA (approximately 100 ng). The reaction was adjusted to the total volume of 25 μl by double-distilled water (ddH2O).

The PCR was performed using an initial denaturation step at 94 °C for 5 minutes, followed by 30 cycles of denaturation at 94 °C for 30 seconds, annealing at 58 for 30 seconds, and elongation at 72 °C for 30 seconds. PCR products were examined by 1% agarose gel electrophoresis for the presence and sizes of amplicons.

Consequently, DNA sequencing of the PCR products was performed on 3130 ABI capillary electrophoresis using forward and reverse primers for each amplicon. Sequencing chromatograms were analyzed by using CodonCode aligner software.

The allele frequencies of the identified variants in *ATP7B* gene were determined by using the following public databases: dbSNP Common 144 of the National Center for Biotechnology Information (NCBI), 1000 Genomes Project phase 3 of The International Genome Sample Resource (IGSR; www.1000genomes.org), Exome Aggregation Consortium (ExAC) version 0.3, and the Iranian Genome Project (https://irangenes.com/data-2/).

*In silico* predictions of the pathogenicity of the identified variants were performed using the following software: SIFT (http://sift.jcvi.org/), PolyPhen-2 (http://genetics.bwh.harvard.edu/pph2/), Mutation Taster (http://www.mutationtaster.org/), and PROVEAN human genome variants (http://provean.jcvi.org/genome_submit_2.php). Finally, conservation of the region harboring the mutations was surveyed by comparing these regions of the genome in the Human, Dog, Rhesus, Mouse, Elephant, Chicken, and some other vertebrates in UCSC and ConSurf databases [20].

In order to check the allele frequencies of c.1924G>C and c.3809A>G variants in 100 healthy ethnicity-matched controls, tetraplex amplification-refractory mutation system (ARMS)-PCR was employed. The primers and size of expected bands in each reaction are depicted in Table 2.

**Table 2:** Amplification-refractory mutation system primer sequences used for checking c.1924G>C and c.3809A>G variants in healthy populations controls

	Primer name	Sequence	Expected size of product fraction
c.1924G>C	EX6F_inn	5'-CCCAACGCTCATCACTTAG-3'	173 bp
	EX6R_inn	5'-GCTTTATTTCCATCTTGTGGAG-3'	237 bp
	EX6F_out	5'-CTATTGGGTAAAGAAGTTGTAAGCAG-3'	369 bp
	EX6R_out	5'-ATTACAAGGGTAAAGGCAGCTAAT-3'	
c.3809A>G	EX18F_in	5'-GTGGGGGATGGGGTCTG-3'	289 bp
	EX18R_in	5'-CAAGGCCGGGGAGTCCT-3'	196 bp
	EX18Fout	5'-GTAACTTGAGGTTTCTGCTGCTAT-3'	451 bp
	EX18Rout	5'-AGGTTATAAATCAGTGCCAGGAC-3'	

In sample IV:5, who was the proband of this family, sequencing of all exons and exon–intron boundaries of *ATP7B* gene revealed seven alterations which are presented in Table 3. Among these variants, only the allele frequencies of c.1924G>C and c.3809A>G were less than 0.01 in the 1000 Genomes Project and other public databases. The rest of the variants were common and had allele frequencies more than 0.01. For example, there were no homozygote cases in ExAC database for these two variants and the heterozygote rate was also very low (0.0001491 and 8.281e-06 respectively). Therefore only these two variants were considered for further investigations.

**Table 3:** List of all variants found in *ATP7B* gene by sequencing of all the exons and exon–intron boundaries in sample IV:5

Nucleic acid alteration	Amino acid alteration	Location of gene	Zygosity	Chr. location	RS ID	1000 Genomes freq
c.1924G>C	p.Asp642His	EX06	Het	chr13:52,535,995	rs72552285	0
c.3809A>G	p.Asn1270Ser	EX18	Het	chr13:52,511,706	rs121907990	0
c.3903 + 6C>T	–	IN18	Het	chr13:52,511,606	rs2282057	0.4753
c.3419 T>C	p.Val1140Ala	EX16	Het	chr13:52,515,354	rs1801249	0.4652
c.3009G>A	p.Ala1003Ala	EX13	Het	chr13:52,520,471	rs1801247	0.0568
c.2855G>A	p.Arg952Lys	EX12	Het	chr13:52,523,808	rs732774	0.4725
c.2495A>G	p.Lys832Arg	EX10	Het	chr13:52,524,488	rs1061472	0.4753

*chr* chromosome, *freq* frequency, *RS ID* reference SNP cluster ID

The c.1924G>C variant is inside exon 6 of *ATP7B* gene at the position of chr13:52,535,995 and is a missense point mutation (5′**G**AC→**C**AC3′) which results in substituting aspartic acid (D) codon to histidine (H) aa at the residue of 642 in the translated protein sequence. This variant has been reported in dbSNP as rs2552285.

The c.3809A>G variant affects exon 18 of *ATP7B* gene at the position of chr13:52,511,706 (5′A**A**T→A**G**T3′) causing asparagine (N) aa codon change to serine (S) at the residue of 1270 in protein level. This variant has been reported also in dbSNP as rs121907990.

**Fig. 2:**
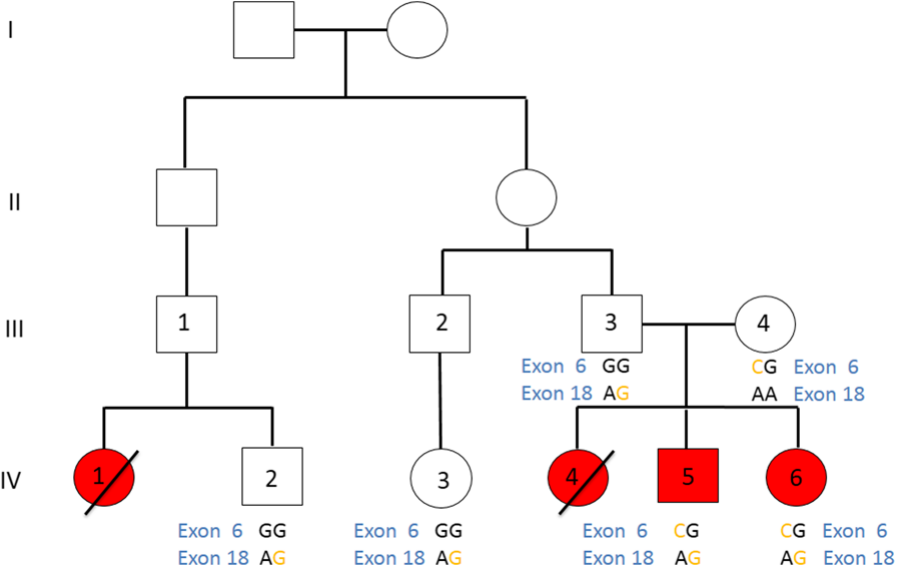
Genotypes of the c.3809A>G and c.1924G>C mutations in *ATP7B* gene in studied healthy and affected individuals of the pedigree

**Fig. 3:**
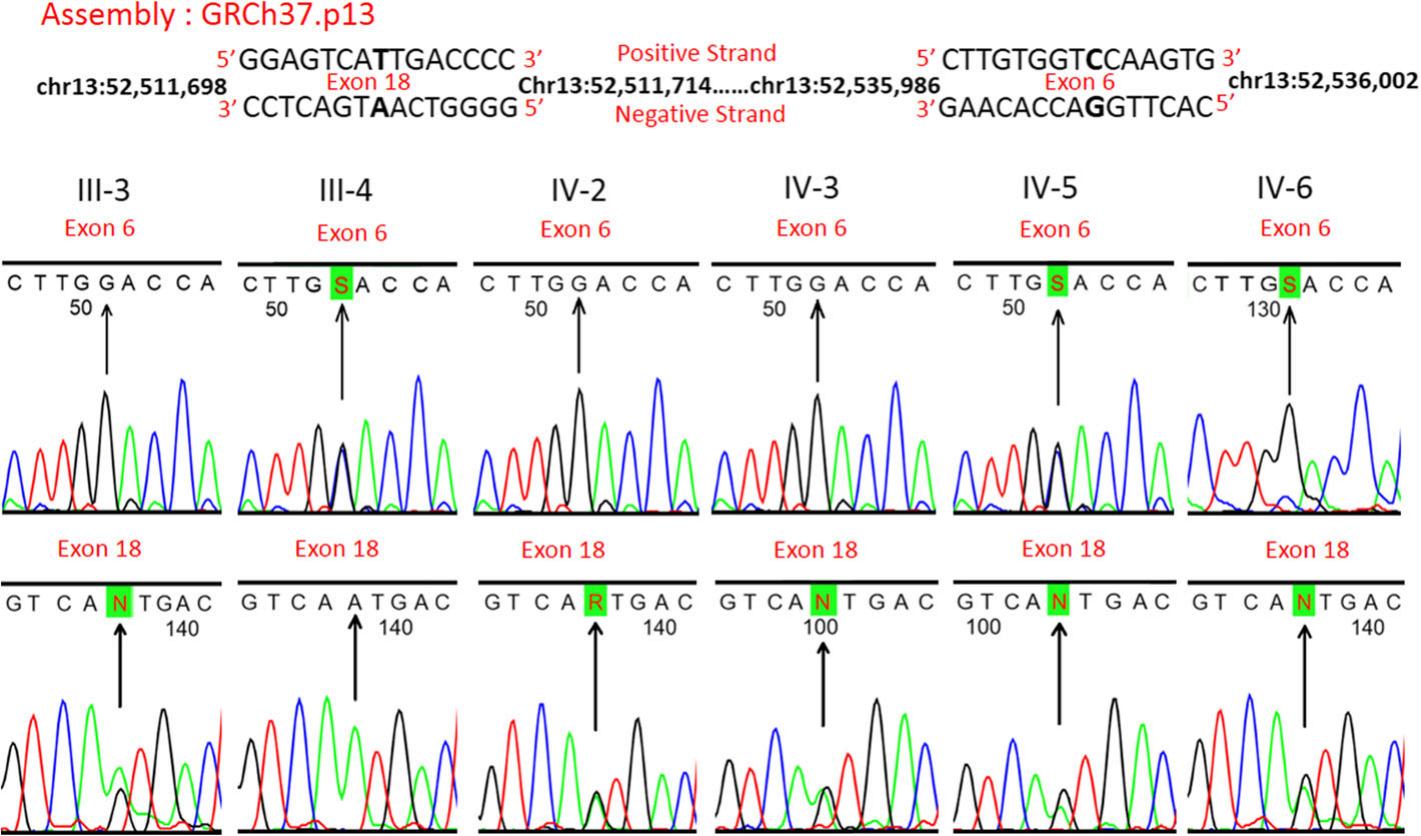
Sanger sequencing traces showing the c.1924G>C; p.Asp642His mutation in exon 6 (*upper row*) and c.3809A>G; p.Asn1270Ser mutation in exon 18 (*lower row*) of the *ATP7B* gene. The segregation of these two mutations as compound heterozygote has been confirmed in six available DNA samples (two affected and four unaffected individuals) from this family

The segregation of these two variants in the family was checked by Sanger sequencing on all available samples (Figs. 2 and 3). The segregation study indicated that the c.3809A>G; p.Asn1270Ser and c.1924G>C; p.Asp642His mutations in *ATP7B* gene are co-segregating with the disease in this family. Affected members of the family were compound heterozygote for these mutations and each of the parents carried only one of the defective alleles.

**Fig. 4:**
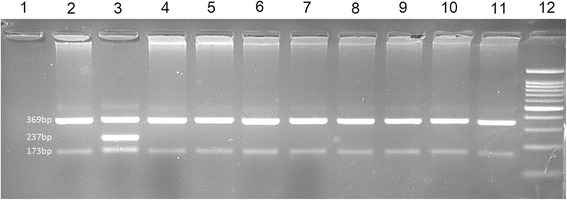
Picture of 2% agarose gel electrophoresis of amplification-refractory mutation system test products for exon 6 variant (c.1924G>C). Well 1: negative polymerase chain reaction test control (no template control; NTC). Well 2: normal case homozygote for GG. Well 3: IV:5 member in the family who was heterozygote for c.1924G>C (GC). Well 4–11: eight healthy members. Well 12: 100 kb ladder

**Fig. 5:**
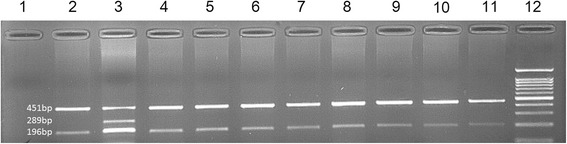
Picture of 2% agarose gel electrophoresis of amplification-refractory mutation system test products for exon 18 variant (c.3809A>G). Well 1: negative polymerase chain reaction test control (no template control; NTC). Well 2: normal case homozygote for AA. Well 3: IV:5 member in the family who was heterozygote for c.3809A>G (AG). Well 4–11: eight healthy members. Well 12: 100 kb ladder

We checked 100 healthy controls for both variants in exons 6 and 18 using ARMS method and the acquired results showed they were negative (Figs. 4 and 5).

*In silico* analysis of these two variants by Mutation Taster, SIFT, Polyphen-2, and PROVEAN software predicted that these two variants are pathogenic and they have damaging effects on the protein function.

**Fig. 6:**
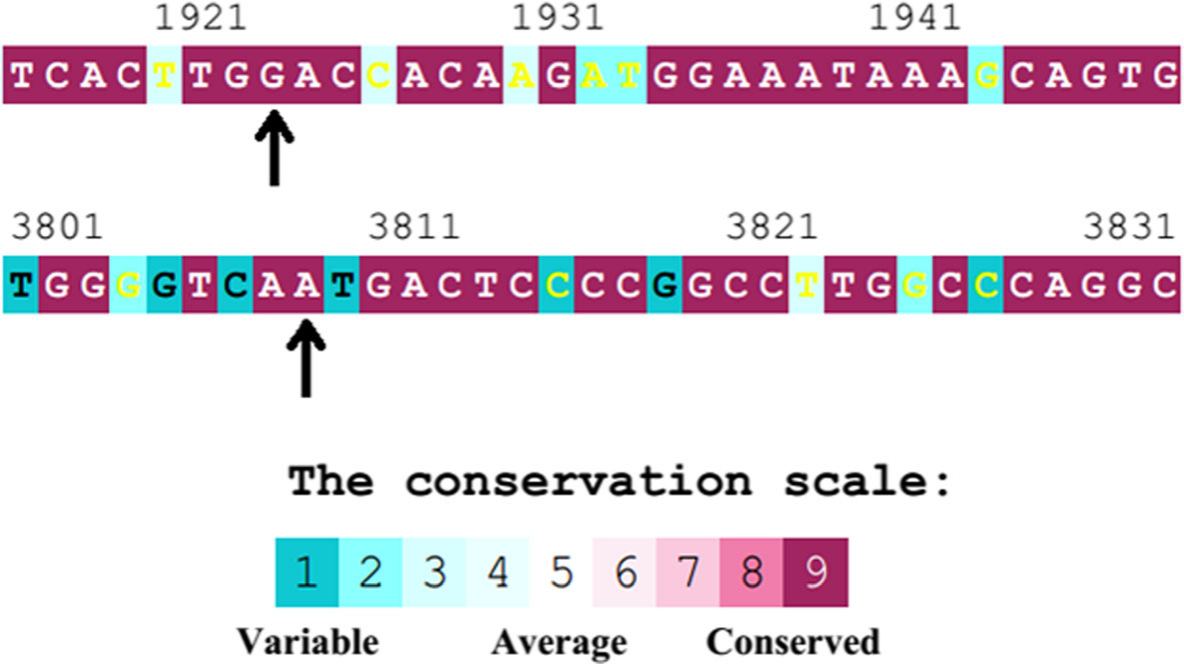
The conservation scores for the amino acids at the region of p.Asn1270Ser and p.Asp642His mutations in *ATP7B* gene calculated by ConSurf tool. ConSurf estimates the evolutionary conservation of amino acid residues in a peptide based on the phylogenetic relations between homologous sequences as well as amino acid’s structural and functional importance

In addition, multiple alignments of the region of these two mutations in several species such as Human, Dog, Rhesus, Mouse, Elephant, and Chicken in UCSC and ConSurf tool showed that they are highly conserved arguing that these residues play an important role in the function of this protein (Fig. 6).

## Discussion

After analyzing all affected and healthy members available in this pedigree, a compound heterozygote pattern of inheritance in this family was confirmed, which is not considered to be a common phenomenon of WD mutations in the Iranian population [21].

In this study we examined mutations in the coding regions of the *ATP7B* gene in an Iranian family which resulted in the identification of two mutations. A cohort of 100 ethnicity-matched healthy controls turned out to be negative for these two mutations as well as the 1000 Genomes and ExAC databases.

*In silico* analysis of the two mentioned mutations with ConSurf software showed high conservation among vertebrate species of these residues.

The first mutation (p.Asp642His) found in this pedigree is located in the cytoplasmic region at 64 aa after the last copper binding domain. This mutation transforms a negatively charged residue (Asp) to a positively charged one (His). Presumably this mutation by affecting the domain affinity to copper or the folding structure in the cytoplasmic region decreases the stability of this domain, and this can lead to abnormal localization of the protein within cytoplasm and impairment of protein function.

**Fig. 7:**
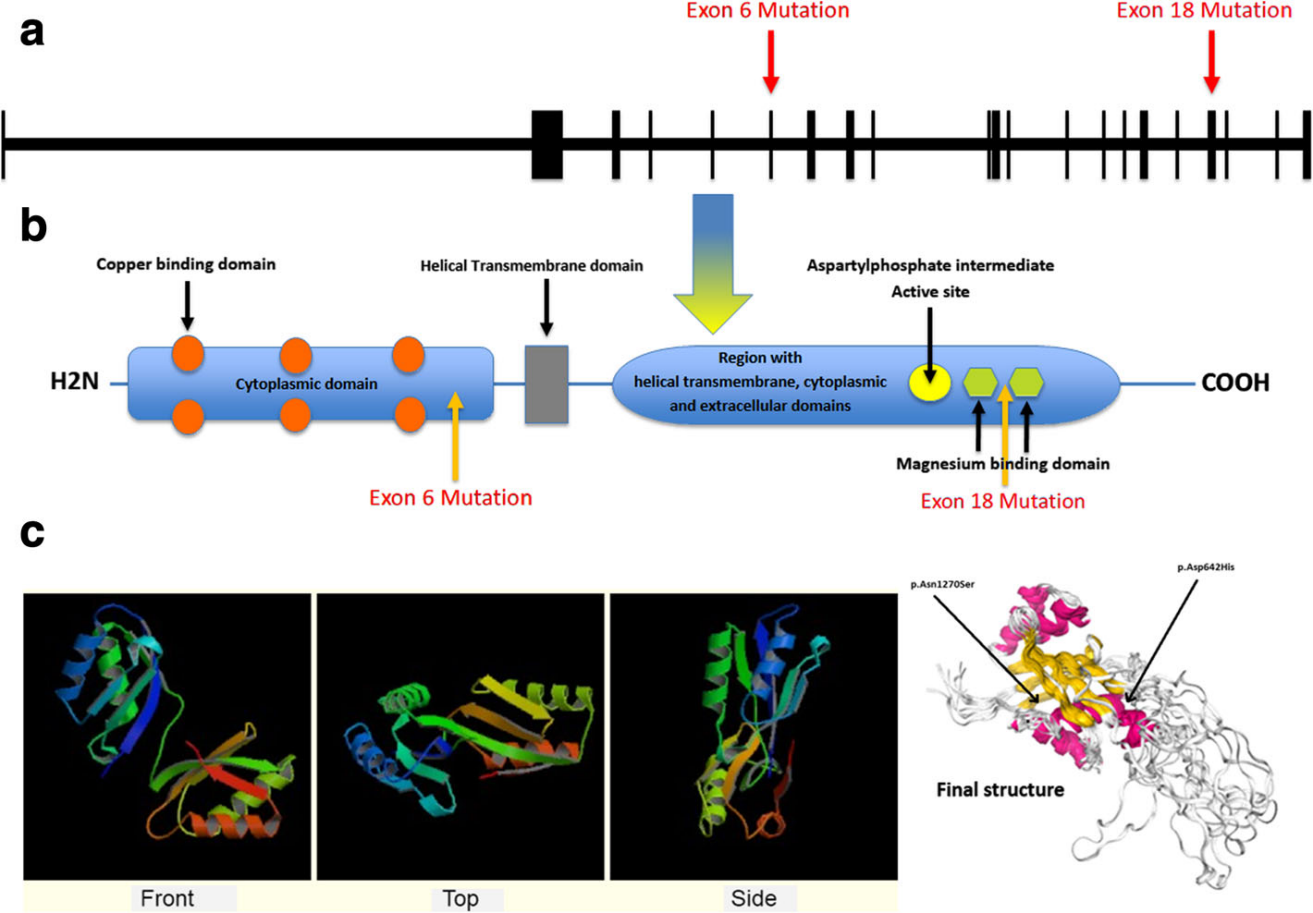
Schematic presentation of *ATP7B* gene structure in DNA (**a**) and protein levels (**b**). Location of the c.3809A>G; p.Asn1270Ser and c.1924G>C; p.Asp642His mutations in *ATP7B* gene are depicted according to their relevant domains (**c**). Three-dimensional structure of ATP7B protein and the coordination of mutations are depicted

The second mutation (p.Asn1270Ser) also affects the cytoplasmic region of this protein at one aa before the second magnesium binding domain region of ATP7B protein (1271 residue), transforming a nonionic polar aa (Asn) to another nonionic polar one with the different side chain (Ser). Presumably this mutation affects the magnesium binding affinity and domain structure of the protein. A schematic presentation of the *ATP7B* gene structure in DNA and protein levels, as well as the three-dimensional structure of the ATP7B protein and the coordination of mutations are depicted in Fig. 7.

WD mutation frequency and types are different among different populations. For example, c.2333G>T in exon 8 is the commonest mutation in the Korean population [22]. The c.3207C>A mutation (H1069Q) in exon 14 is the commonest mutation in Turkish and Iranian populations, which is also shown to be a common mutation in the European population [6, 21]. The c.3207C>A mutation has been shown to be associated with late onset neurological conditions in European countries, whereas in the one cohort study in the Iranian population it has been observed in patients who all had hepatic symptoms and were 5 to 40-years old [21]. Exon 18 is reported as a hotspot exon in Western countries while the hot spot exons reported in China were 8 and 12. In addition, some populations, such as the Egyptian population, have shown very heterogeneous allele frequencies for WD mutations in their populations with respect to their ethnicities [23].

The attractive dimension in diseases like WD – that the defective gene in the disease encodes a protein with several distinct domains and any of them play a certain role in protein function and phenotype characteristic – is that: differences in the type of mutation and its place on the protein structure may result in different symptoms and clinical outcomes due to the location of the mutation in the protein structure and severity of the mutation.

It has been reported that mutations in the transmembrane domain and ATP loop result in early onset of disease (> 8 years); furthermore, ATP loop mutations tend to cause hepatic symptoms with absence of neurological symptoms. Mutations in ATP hinge result in hepatic failure, and transmembrane and copper binding mutations are associated with neurological manifestations. Finally, there have been more reports of frameshift mutations in patients with early hepatic manifestation, whereas splice site mutations have been reported in patients with neurological phenotypes [23].

It seems the missense mutations on cytoplasmic region result in hepatic impairment with late onset of neurological manifestations, like what was experienced by affected members in the family studied here, but this observation needs large-scale analysis in bigger cohorts to confirm the genotype-phenotype correlation.

## Conclusions

In this study we showed that even in a population like Iran with a high rate of inbreeding it is still possible to find recessive disorders like WD as a result of compound heterozygote mutations in a pedigree with no recent consanguinity. Therefore heterozygote mutations should also be considered both in affected and carrier members of high-risk families.

## Abbreviations

AR: Autosomal recessive
ARMS: Amplification-refractory mutation system
dbSNP: Single Nucleotide Polymorphism database
ExAC: Exome Aggregation Consortium
PCR: Polymerase chain reaction
UCSC: University of California, Santa Cruz
WD: Wilson disease
